# Corrigendum

**DOI:** 10.1002/rmb2.12035

**Published:** 2017-04-18

In^1^ the following error was published on page 181 under the heading “Patients’ characteristics” of “Results”.

The mean ages of the groups were not statistically significantly different.

The text was incorrect and should have read:

The mean ages of the groups were statistically significantly different.

The authors apologize for this error.
